# Reactivation of Tuberculosis in the Setting of COVID-19 Infection

**DOI:** 10.7759/cureus.23417

**Published:** 2022-03-23

**Authors:** Muhammad Atif Masood Noori, Islam Younes, Asnia Latif, Hardik Fichadiya, Sherif Elkattawy, Harshwardhan Khandait, Onyeka Nawachukwu, Vipin Garg

**Affiliations:** 1 Internal Medicine, Rutgers Health/Trinitas Regional Medical Center, Elizabeth, USA; 2 Internal Medicine/Pulmonology, Rutgers Health/Trinitas Regional Medical Center, Elizabeth, USA

**Keywords:** corticosteroids, lymphopenia, cavitary lung lesion, tb, covid-19 pneumonia

## Abstract

Coronavirus disease 2019 (COVID-19) caused by severe acute respiratory syndrome coronavirus 2 (SARS-CoV- 2) was declared a pandemic by WHO in March 2020. The causative organism has since undergone a series of mutations. COVID-19 primarily being a respiratory illness causes pre-existing pulmonary diseases to show worse clinical outcomes.

About one-third of the world’s population is thought to be infected with latent *Mycobacterium tuberculosis* (MTB). Both previous and newly developed tuberculosis (TB) infection are risk factors for COVID-19 and are associated with poor outcomes. T lymphocytes play a pivotal role in defense against MTB and with evidence suggesting depletion of T lymphocytes in COVID-19, it can be postulated that COVID-19 can increase the risk of reactivation of latent TB.

Given that a large population around the globe is infected with latent tuberculosis, it is interesting to study and note cases where the virus leads to the reactivation of latent tuberculosis infection. Herein, we present a 76-year-old Brazilian male recently treated for COVID-19 pneumonia, presenting with new-onset cough and weakness diagnosed with latent MTB reactivation.

## Introduction

Since the coronavirus disease 2019 (COVID-19) pandemic outbreak, multiple studies have been conducted to study the disease better. Although the virus is notorious to cause severe acute respiratory distress syndrome, it is also known to cause other pulmonary and extra-pulmonary manifestations. Pulmonary complications include pulmonary fibrosis and increased incidence of thromboembolic events [1,2]. Extra-pulmonary manifestations include nausea, vomiting, diarrhea, elevation in liver enzymes, myocarditis, pericarditis, cardiogenic shock, acute kidney injury, dizziness, headaches, stroke, psychosis, uveitis, conjunctivitis, and skin rashes [3].

Patients with pre-existing pulmonary disease are at a higher risk of severe COVID infection [4]. COVID-19 causing an immuno-suppressive state can cause prone patients to have reactivation of latent infection. Given that a large population around the globe is infected with latent tuberculosis, it is interesting to study and note cases where the virus leads to the reactivation of latent tuberculosis infection. 

## Case presentation

**Figure 1:**
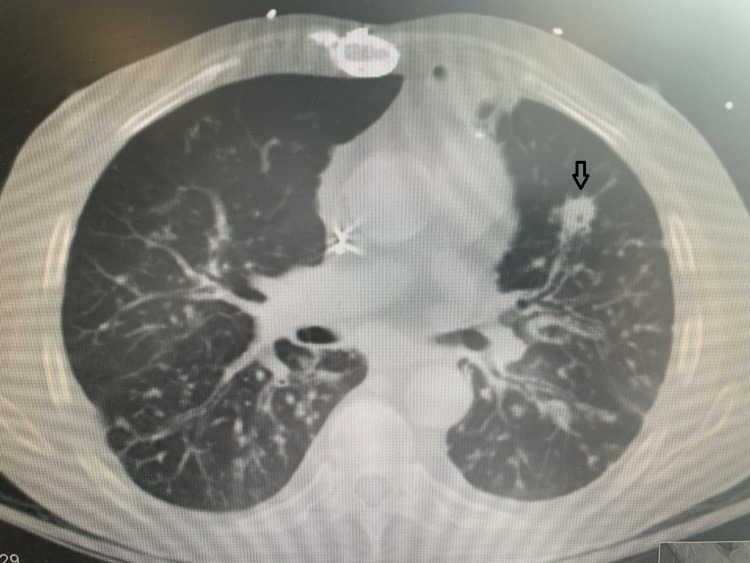
CT chest without contrast The arrow shows extensive patchy, and tree-in-bud opacities with a few of them demonstrating internal cavitation

A 76-year-old Brazilian male with a past medical history of chronic obstructive pulmonary disease, coronary artery disease, seizure disorder, and heart failure with reduced ejection fraction presented to the emergency department with complaints of generalized weakness associated with non-productive cough. One month prior to the presentation, he had a mild COVID-19 infection that did not require hospitalization. The patient denied any fever, chest pain, shortness of breath, abdominal pain, or any changes in urinary or bowel habits. On arrival, his blood pressure was 110/70 mmHg, pulse rate was 100 beats per minute, respiratory rate was 20 breaths per minute, temperature 98.1 F, and oxygen saturation of 94% on 4 liters nasal cannula. Laboratory values were as follows: WBC 9800/µL, Hemoglobin 10.4 g/dL, Platelet 512 × 10^3^/µL, Troponin 0.06 ng/mL, brain natriuretic peptide (BNP) 117, creatinine 0.86 mg/dL, urea nitrogen 11 mg/dL. Chest X-ray showed bilateral reticulonodular and patchy opacities with left apical and basilar scarring. CT chest without contrast demonstrated extensive patchy, peribronchiolar, and tree-in-bud opacities with a few of them demonstrating internal cavitation (Figure 1).

Extensive infectious workups including blood, respiratory, and urine culture, interferon-gamma release assay (IGRA), and multiple sputum samples for acid-fast bacilli (AFB) were sent. The patient was maintained in airborne precautions and empirically treated with intravenous (IV) ceftriaxone and azithromycin. Quantiferon TB Gold assay was positive, however, multiple sputum AFB tests were negative. Bronchoscopy with lavage and trans-bronchial biopsy of the left upper lobe was also performed. Cytology of lavage demonstrated acute inflammatory cells, bronchial epithelial cells, and macrophage. It was also positive for AFB. Bronchial washing polymerase chain reaction (PCR) test also came out positive for MTB complex and patient was started on isoniazid, rifabutin, pyrazinamide, and ethambutol. The patient was maintained in isolation for at least 2 weeks of therapy and then was discharged to follow up TB clinic as an outpatient. 

## Discussion

TB is caused by MTB. It is one of the most feared contagious infectious diseases, especially, in low-income countries. In 2020, an estimated 10 million people contracted and became ill with TB and a total of 1.5 million people died in the same year [5]. It primarily affects the lungs but can affect almost any organ in the body via hematological seeding. In a fraction of cases, the host can either mount an immune response strong enough to destroy the bacterium or it can manifest as an active disease. However, in most cases, it causes latent infection, meaning the bacterium replicates in the macrophages and initiates the inflammatory host response that walls the infection off in a granuloma, preventing the manifestation of clinical symptoms. These granulomas contain the disease for years. Dysregulation of the host immunity can re-activate latent TB (LTB) [6].

With the emergence of the COVID-19 pandemic, there have been numerous reports on the rates of incidence of TB. A few studies report that there has been an overall decrease in the contraction of TB since the COVID-19 pandemic because people are practicing social distancing and more aggressive hygiene routine like hand washing and sanitizing [7]. However, because initially people had been advised to stay at home, there was a surge in the household spread of TB owing to prolonged exposure to the infected person [8].

LTB is known to be reactivated by any factors causing immunosuppression including HIV infection, organ transplantation, immunosuppressant drugs, silicosis, and kidney dialysis [9]. As per the RECOVERY trial treatment, the use of high-dose steroids reduced the 28-day mortality rate in COVID-19 patients requiring some sort of respiratory support [10]. COVID-19, like TB, is also a respiratory infection and induces a robust inflammatory response in the host. While the controlled inflammatory reaction may protect the body against viral infections, an uncontrolled cytokine storm, as seen in COVID-19, can dysregulate the host immune system which in turn leads to acute respiratory distress syndrome (ARDS) and worsening of clinical symptoms [11]. Corticosteroids act on intranuclear glucocorticoid response elements and regulate the expression of various genes. They reduce the inflammatory process by upregulating the anti-inflammatory molecules like interleukin (IL)-10 and inhibiting the proinflammatory cytokines like phospholipase A2. Moreover, they hamper the host reactions like chemotaxis and extravasation of immune cells. On a molecular level, steroids induce glucocorticoid-induced leucine zipper (*GILZ*) which is an anti-inflammatory gene. *GILZ* prevents NF-kB activation which inhibits the activation of the mitogen-activated protein (MAP) kinase pathway, thus inhibiting inflammatory response [12]. While all these anti-inflammatory properties are beneficial, steroids inadvertently also cause immunosuppression by sequestration of CD4+ T lymphocytes [13]. In COVID patients treated with steroids, therapeutic immunosuppression can lead to the reactivation of LTB. In addition, FDA-approved monoclonal antibodies like tocilizumab, bamlanivimab, and estesevimab for the treatment of COVID-19 in early stages can directly cause re-activation of TB [14-16].

The lymphopenia caused by COVID-19 itself increases the chances of LTB activation. There is a murine model studied for coronavirus-induced reactivation of TB. This study revealed 20 times lower viral load in mice with dormant MTB. The defense mechanism involves reprogramming of CD271 and mesenchymal stem cells which activates the stemness genes leading to intracellular MTB replication and extracellular release [17].

Conversely, the chances of COVID-19 contraction in patients diagnosed with TB are also reported to be increased compared to the general population. The simple explanation is that baseline lung disease and the illness itself make patients more prone to having COVID-19 and more serious consequences of the disease.

## Conclusions

Tuberculosis infection should be suspected in patients with a history of COVID-19 pneumonia, presenting with cavitary lung lesions. The objective predictor of the possibility of reactivation of LTB in COVID-19 cases is yet to be investigated. We propose that there should be more studies to investigate the effects of COVID-19 infection on the reactivation of TB. 
